# Megaloptera of Canada

**DOI:** 10.3897/zookeys.819.23948

**Published:** 2019-01-24

**Authors:** Xingyue Liu

**Affiliations:** 1 Department of Entomology, China Agricultural University, Beijing 100193, China China Agricultural University Beijing China

**Keywords:** alderflies, biodiversity assessment, Biota of Canada, dobsonflies, fishflies, Megaloptera

## Abstract

An updated summary on the fauna of Canadian Megaloptera is provided. Currently, 18 species are recorded in Canada, with six species of Corydalidae and 12 species of Sialidae. This is an increase of two species since 1979. An additional seven species are expected to be discovered in Canada. Barcode Index Numbers are available for ten Canadian species.

The order Megaloptera (dobsonflies, fishflies, and alderflies) is one of the three orders of Neuropterida, and is characterized by the prognathous adult head, the broad anal area of hind wing and the exclusively aquatic larval stages (New and Theischinger 1993). Currently, there are ca. 380 described species of Megaloptera worldwide (Yang and Liu 2010, Oswald 2016). Extant Megaloptera are composed of only two families; Corydalidae, which is divided into Corydalinae (dobsonflies) and Chauliodinae (fishflies), and Sialidae (alderflies). Major species diversity of Megaloptera is confined to the subtropical and warm temperate regions, e.g., the Oriental, Neotropical, and Australian Regions (Yang and Liu 2010, Liu et al. 2012, 2015).

Southern Canada is probably the northern limit of the distribution range of Nearctic Megaloptera. Recent phylogeographic studies suggest that the Canadian population of a fishfly species (i.e., *Nigroniaserricornis* (Say)) was formed very rapidly after the Pleistocene glacial period (Heilveil and Berlocher 2006). Thus, the Canadian Megaloptera may play an important part in understanding the evolutionary history of Nearctic Megaloptera.

All known Canadian species of Megaloptera are also found in the United States of America. Kevan (1979) recorded 16 native species of Megaloptera in Canada, including six species of Corydalidae and 10 species of Sialidae. Since then, no more species of Corydalidae have been found in Canada. Evans (1984) reported two new fishflies, *Orohermescrepusculus* (Chandler) and *Protochauliodescascadius* Evans, from the northwestern USA where they coexist with *Dysmicohermesdisjunctus* (Walker) and *Protochauliodesspenceri* Munroe in Oregon (Evans 1972). As the latter two species are known from southwestern British Columbia, *O.crepusculus* and *P.cascadius* may also be found in similar habitats there. Additionally, *Neohermesconcolor* (Davis), which is widespread in the eastern USA (Liu et al. 2016), may also occur in adjacent areas of Canada. It should be also noted that in the Species Catalogue of Lacewing Digital Library (LDL), the most comprehensive and frequently updated database of species of Neuropterida (Oswald 2016), *Chauliodesrastricornis* Rambur is not recorded in Canada, although the Canadian record of this species is reported by van der Weele (1910). Herein the distribution of *C.rastricornis* in Canada is confirmed.

For Canadian alderflies, the most important faunal work after Kevan (1979) is that of Whiting (1991) in which *Sialisinfumata* Newman and *S.joppa* Ross were newly recorded in Canada. An additional four species of *Sialis* are estimated to be found in Canada, namely *S.aequalis* Banks, *S.driesbachi* Flint, *S.hasta* Ross, and *S.spangleri* Flint, because these species are distributed around the Great Lakes region of northeastern USA that is in close promimity to Canada.

DNA barcodes are available for all but one species of known Canadian Corydalidae, but for less than half of Sialidae (Table 1). Species with DNA barcodes comprise *Chauliodespectinicornis* (Linnaeus) (BOLD:AAH3593), *C.rastricornis* (BOLD:AAH3594), *D.disjunctus* (BOLD:ACA3660), *N.serricornis* (BOLD:AAA1274), *P.spenceri* (BOLD:ACP8653), *Sialisconcava* Banks (BOLD:AAL6477), *S.hamata* Ross (BOLD:ACA3407), *S.joppa* Ross (BOLD:AAG9766), *S.vagans* Ross (BOLD:AAH7456), and *S.velata* Ross (BOLD:AAG9765). Current barcode information does not indicate the presence of cryptic species.

**Table 1.** Census of Megaloptera in Canada.

Taxon^1^	No. species reported in Kevan (1979)	No. species currently known from Canada	Number of BINs^2^ available for Canadian species	Est. no. undescribed or unrecorded species in Canada	General distribution by ecozone^3^	Information sources
Corydalidae	6	6	5	3	Boreal Shield, Pacific Maritime, Atlantic Maritime, Mixedwood Plains	van der Weele 1910, Evans 1972, Yang and Liu 2010, Oswald 2016, Liu and Winterton 2016
Sialidae	10	12	5	4	Boreal Shield, Boreal Plains, Pacific Maritime, Atlantic Maritime, Montane Cordillera, Mixedwood Plains, Prairies	Whiting 1991, Liu et al. 2015
**Total**	**16**	**18**	**10**	**7**		

^1^Classification follows that of Yang and Liu (2010). ^2^Barcode Index Number, as defined in Ratnasingham and Hebert (2013). ^3^See figure 1 in Langor (2019) for a map of ecozones.

Thirteen of the 18 species of Canadian megalopterans have their larval stage described, and their life history is known (Davis 1903, Cuyler 1958, Neunzig 1966, Azam and Anderson 1969, Evans 1972, Leischner and Pritchard 1973, Lilly et al. 1978); however, most of the information is based on studies of materials or populations from the United States.

Additional surveys of Megaloptera habitats in southern Alberta and British Columbia, especially southwestern British Columbia, are warranted to fill in gaps in distribution and to ascertain whether other species are present. Fresh material of all megalopterans, especially Sialidae, is needed for obtaining DNA barcodes.
